# Augmented immune responses to a booster dose of oral cholera vaccine in Bangladeshi children less than 5 years of age: Revaccination after an interval of over three years of primary vaccination with a single dose of vaccine

**DOI:** 10.1016/j.vaccine.2019.12.034

**Published:** 2020-02-11

**Authors:** Fahima Chowdhury, Taufiqur Rahman Bhuiyan, Afroza Akter, Md Saruar Bhuiyan, Ashraful Islam Khan, Imam Tauheed, Tasnuva Ahmed, Jannatul Ferdous, Pinki Dash, Salima Raiyan Basher, Al Hakim, Julia Lynch, Jerome H. Kim, Jean-Louis Excler, Deok Ryun Kim, John D. Clemens, Firdausi Qadri

**Affiliations:** a International Centre for Diarrhoeal Disease Research Bangladesh (icddr,b), Dhaka, Bangladesh; b International Vaccine Institute (IVI), Seoul, Republic of Korea; c UCLA Fielding School of Public Health, Los Angeles, CA, USA; d Korea University School of Medicine, Seoul, Republic of Korea

**Keywords:** Oral Cholera Vaccine (OCV), Augmented immune response, Booster dose, Primary dose, Shanchol

## Abstract

We have earlier reported that a single dose of oral cholera vaccine (OCV) is protective in adults and children ≥5 years of age and sustained for 2 years. We enrolled participants (n = 240) from this study, between March-September 2017, over 3 years after receiving a primary single dose. Immune responses were measured in placebo group (Primary Immunization group: PI) and compared with those who received a single dose (Booster Immunization group: BI). The children were 4 to <5 years, 5 to <18 years and adults >18 years. Blood was collected at day 0 (before vaccination) and after receiving 1st and 2nd doses of OCV. Overall, the BI and PI groups showed vibriocidal antibody response after 1st and 2nd dose of vaccination in all age groups to *V. cholerae* O1 and O139. Young children in the BI group showed significantly higher vibriocidal antibody response two weeks after receiving the first dose as compared to PI group to LPS. Elevated plasma IgA responses to LPS after the first dose were observed among the BI group compared to the PI group among the young children. Mucosal antibody responses measured in fecal extracts showed similar increases as that of vibriocidal and LPS responses in the BI group. These results suggest a single boosting dose of OCV generated immune response in primed population >5 years of age who had earlier received OCV. However, young children who had received OCV earlier, boosting after a single dose, resulted in increased immune responses compared to the PI group. Further studies are needed to assess protection obtained from different strategies, especially for young children and to determine the numbers of primary and booster doses needed. In addition, more information is needed regarding the optimum interval between primary and booster doses to plan future interventions for cholera control.

ClinicalTrials.gov Identifier: NCT 02027207.

## 1 Introduction

Cholera remains a major public health concern despite years of control efforts, causing an estimated 1.3–4.0 million cases and 21,000–143,000 deaths per year [1]. Cholera affects primarily people in low- and middle-income countries (LMIC) but also fragile populations in humanitarian crises with poor access to adequate water and sanitation resources. At a global level, prevention focuses on the use of oral cholera vaccines (OCV) to control the disease particularly in outbreaks but also epidemics in endemic settings where rapid action is required [1]. In 2017, the Global Task Force on Cholera Control (GTFCC) launched a strategy aimed at reducing cholera deaths by 90% and eliminating cholera in as many as 20 countries by 2030 [2].

Although two doses of OCV are recommended for protection against cholera, a single dose of OCV can also prevent cholera cases reducing logistical requirements [1], [3]. Several immunogenicity studies with OCV suggest that a single dose of oral cholera vaccine elicits an antibody response similar to that seen after two doses given 2–4 weeks apart [4], [5]. Epidemiological studies have shown that a single dose of OCV provided moderate protection from cholera [3], [6], [7]. In earlier studies, we reported that after intake of two doses of OCV given at 14 days intervals, vibriocidal antibody responses rates against *V. cholerae* O1 Inaba, Ogawa; and O139 serotypes were 60%, 72% and 21% in adults, 84%, 75% and 64% in toddlers, and 74%, 78% and 54% in young children [8]. Another study conducted in Kolkata showed an OCV boosting effect five years after the primary series which elicited an increased vibriocidal immune response among children [9]. However, vaccine efficacy was lower among children less than 5 years of age [10]. In the present study we have evaluated the booster effect on the immune responses among different age groups of children and adults who received a single dose of OCV (Shanchol™) three years earlier and were then given two doses of OCV, 14 days apart.

In 2014, we conducted a randomized, double-blind, placebo-controlled trial in cholera-prone urban slums of Dhaka, Bangladesh, to estimate the protection to a single dose of OCV. The protective efficacy was 39% against all cholera episodes and 50% against cholera with severe dehydration in the 2 year follow-up. The vaccine protective efficacy was 57% in individuals’ ≥5 years of age. However, in participants <5 years of age, the single dose of vaccine did not show protection against cholera [11], although the study was underpowered to analyze the level of protection [12]. Similar to this finding, a systematic review of OCV efficacy has revealed reduced protection among young children compared to individuals >5 years of age following intake of the recommended two doses of OCV [13]. The lower vaccine efficacy after a single dose in children is low even after 6 months [7] and is of concern and prompted us to evaluate the immunogenicity of two additional doses of vaccine administered three year later after the initial OCV single dose. A group of participants from the earlier trial were enrolled in the study [11] to evaluate the boosting effect on the immune responses among children and adults who received a single dose of Shanchol or placebo.

## 2 Materials and methods

### 2.1 Study design, participants and eligibility

This was open-label controlled trial conducted among 240 healthy non-pregnant participants aged 4–<5 years, 5–<18 years and 18 years and above who were initially vaccinated with a single dose of OCV (Shanchol) or placebo over 3 years earlier in Mirpur, Dhaka as part of a large-scale, placebo-controlled, randomized trial of a single dose of OCV [11]. There were two intervention groups: participants who received OCV over 3 years earlier and were revaccinated with two doses of OCV (boosted immunized (BI) group) and participants who received placebo over 3 years earlier and were vaccinated with two doses of OCV for the first time (primary immunized (PI) group). The two doses of vaccine were given 2 weeks apart between March-September 2017.

Venous blood was drawn at days 0, 3, 14, 17, 28 and 42 to assess vibriocidal antibody titers, B-cell memory response (B-cell response by ELISPOT), and lipopolysaccharide (LPS by ELISA) antibody responses. Corresponding stool samples were also collected for measuring anti-LPS specific IgA antibodies. Participants with chronic illness, any recent illness, with history of diarrheal disease within the last 7 days or with febrile illness in the last 24 hours, who took antibiotics in the last 7 days, pregnant women (identified by the urine strip test of married women) were excluded. Moreover, participants who had any history of confirmed cholera were also excluded. Written informed consent was obtained from adult participants and from guardian of participating children. Assent was also obtained from 11 to 17 years of age participants.

The study was approved by the Institutional Review Boards of icddr,b and of the International Vaccine Institute (IVI). The study was registered with ClinicalTrials.gov number: NCT 02027207.

### 2.2 Study agent and administration

Each dose (1.5 mL) of Shanchol vaccine (produced by Shantha Biotechnics; lot SCN012A16) contained heat-killed and formalin-killed whole-cell bacteria consisting of 600 ELISA Units (EU) of LPS of formalin-killed *V. cholerae* O1 Inaba, E1 Tor biotype (strain Phil 6973) as well as 300 EU LPS of heat-killed *V. cholerae* O1 Ogawa classical biotype (Cairo 50), 300 EU LPS of heat-killed *V. cholerae* O1 Inaba, classical biotype (Cairo 48), and 600 EU LPS of formalin-killed *V. cholerae* O139 (4260B). The vaccinator rotated the vial gently before opening with toothed forceps and gave instructions to the participant to take the oral vaccine.

### 2.3 Follow-up for adverse events following immunization (AEFI)

Participants were requested to wait for at least 30 min at the vaccination sites to monitor any immediate AEFI. Study nurses and physicians involved in vaccine administration were trained for adverse event monitoring. All AEFIs were recorded on a designated form. All side effects were recorded when vaccinated participants attended the field clinic for any medical assistance up to 28 days after each dose of vaccination and reported adverse events were followed up until the event resolved. All adverse events recorded were graded for severity and assessed for possible relatedness to study vaccine by the safety monitor.

### 2.4 Vibriocidal antibody response

Vibriocidal antibody assays were performed using plasma obtained from study participants [14]. Target strains of V. cholerae O1 Inaba (T19479), Ogawa (X25049) and O139 (134B) were incubated with heat-inactivated plasma and exogenous guinea pig complement. Vibriocidal titers were defined as the reciprocal of the highest plasma dilution resulting in a 50% reduction in optical density (595 nm) compared to controls without plasma. To account for inter-assay variation, results were normalized using pooled high titer sera obtained from cholera patients. Positive control sera were used for each plate and assays everyday. The limit of variation to accept the plate was <2 fold of the positive control titre. Seroconversion was defined as a 4-fold or greater increase of vibriocidal titer after vaccination compared to baseline.

### 2.5 LPS ELISA

Enzyme linked immunosorbant assays (ELISA) were performed to assess plasma IgA, IgG and IgM antibody responses to LPS of *V. cholerae* O1 and O139. Ninety six-well plates (Nunc F, Denmark) were coated with V. cholerae O1 Ogawa LPS (2.5 μg/mL) dissolved in phosphate buffered saline (PBS) (pH 7.2–7.4) for anti-LPS [15]. We added 100 μl of plasma (diluted 1:50 for LPS in 0.1% bovine plasma albumin in phosphate-buffered saline–0.05% Tween) per well. Horseradish peroxidase-conjugated secondary antibodies to human IgG or IgA or IgM (Jackson Immunoresearch, West Grove, PA; 1:1000 dilution) were added to the wells, and plates were developed with ortho-phenylene diamine (Sigma, St. Louis, MO) in 0.1 M sodium citrate buffer (pH 4.5) and 0.012% hydrogen peroxide. ELISA plates were read kinetically at 450 nm for 5 min and the maximal rate of change in optical density was measured as milli-absorbance units per minute (mAbs/min).

### 2.6 Memory B-cell (MBC) culture and ELISPOT assay

Memory B-cell (MBC) assays were performed using PBMCs isolated from blood obtained at day 0 before vaccination and at day 42 after vaccination as previously described [16], [17], [18], [19]. We chose to study MBC at the day 42 since we have determined that the B cell responses after OCV intake is optimum at this time point [15], [20]. PBMCs were suspended in RPMI-1640 medium and cultured for 5 days at 37 °C in 5% CO_2_ incubator. B-cell memory was measured by ELISPOT as the percentage of antigen-specific memory B cells out of the total IgA and IgG memory B cells.

### 2.7 Antibody responses in fecal specimens

Mucosal immune responses were evaluated by measuring anti-LPS-specific IgA antibodies in fecal extracts. The total IgA content of each fecal sample was determined by ELISA, as previously described [21]. Fecal antibody levels were determined as the IgA-specific titer divided by the total IgA concentration of each sample. Specimens containing <10 µg/mL or >1000 µg/mL total IgA were excluded from the analysis. As well as samples in which the total IgA concentration varied >3-fold between time points for each participant [22]. These cutoffs were based on previous studies which showed that fecal extracts with either these low or high IgA levels contents in specimens lead to erroneous specific IgA immune responses [21].

### 2.8 Sample size calculation

We calculated sample size of 60 per group in each age category (children and adults) which provided 80% power for a non-inferiority test of GMT ratio between the BI and the PI group, using one-sided test at a 2.5% significance level when the true ratio of the GMT is 1.00. We assumed the non-inferiority margin of 0.5, the coefficient of variation on titer of immunogenicity is 2.0, and the drop-out rate of 10%. Thus, total 240 participants were studied from the BI and PI group.

### 2.9 Statistical analysis

Analysis of demographics and baseline characteristics and safety were analyzed among the participants. The immunogenicity analysis was performed in immunogenicity set which was defined as participants who was received at least one dose of OCV and provide at least one post baseline measure for immunogenicity. In the descriptive analysis, both central tendency (arithmetic mean, median and frequency) and dispersion statistics (range and standard deviation were calculated for participant demographic characteristics and number of adverse events was tabulated among the study groups. For nominal and categorical variables, Fisher’s exact or Chi-square tests and for continuous variable independent two sample *t*-test were performed to see the observed relationship with study groups. Since vibriocidal and anti-LPS antibody responses in plasma specimens may not always follow normal distribution, we reported the geometric mean (GM) as a descriptive statistic instead of arithmetic mean. For comparing the GM among different study groups, we calculated the 95% confidence interval (CI) for each GM and if two CI did not overlap, it was assumed that GMs were significantly different from each other. The ratio of two GMs were tested with reference value 1 to interpret that the GMs were also statistically different. For determining a boosting effect (response), comparison of responses were made between groups (BI and PI) at different study dates after vaccination. Error bar charts were created for presenting GM with CI over the study follow-up days among the study groups. We considered p < 0.05 (two-tailed) as the margin of statistical significance for all test. All the data were entered and managed into Oracle database software by our expert data management team and analyzed using SAS 9.4 (SAS Institute, Cary NC) and Graph Pad Prism 6.0 (GraphPad Software, Inc., La Jolla, CA) software for figures.

## 3 Results

### 3.1 Study participants

**Fig. 1:**
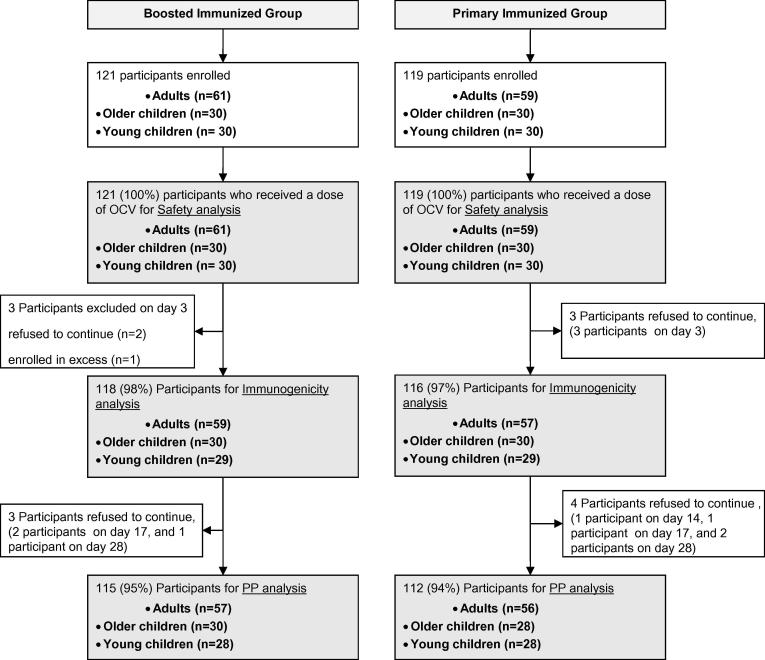
Disposition of study participants.

A total of 240 participants were included in this study, 121 from the BI group and 119 from the PI group. Among them 120 were adults (>18 years; median 32 years), 60 older children (5–18 years; median 8 years) and 60 young children (4–5 years; median 4.7 years). Twelve (5%) participants refused to complete the blood sample collection scheduled at different time points or refused to take the second dose of vaccine. A total of 234 participants (118 in the BI group and 116 in the PI group) who received at least one OCV and provided at least one post baseline measure for immunogenicity were included in immunogenicity analysis. The disposition of participants is shown in Fig. 1. No significant difference was found in age and sex distribution between two groups (Table 1).

**Table 1:** Demographic characteristics of study participants.

Characteristics		Boosted immunized group	Primary immunized group	Total	p-value
Overall		n = 121	n = 119	n = 240	
Gender	Male (%)	60 (49.59)	47 (39.50)	107 (44.58)	0.116
	Female (%)	61 (50.41)	72 (60.50)	133 (55.42)	
Age (years)	Mean (SD)	21.24 (16.95)	20.23 (15.73)	20.74 (16.33)	0.633
	Median (min, max)	18.41 (4.16, 84.42)	17.40 (4.27, 80.04)	17.80 (4.16, 84.42)	

**Adult cohort**		**n = 61**	**n = 59**	**n = 120**	
Gender	Male (%)	28 (45.90)	18 (30.51)	46 (38.33)	0.083
	Female (%)	33 (54.10)	41 (69.49)	74 (61.67)	
Age (years)	Mean (SD)	35.58 (12.08)	33.94 (10.65)	34.78 (11.38)	0.433
	Median (min, max)	33.29 (18.41, 84.42)	31.73 (18.20, 80.04)	32.28 (18.20, 84.42)	

**Older Children cohort**		**n = 30**	**n = 30**	**n = 60**	
Gender	Male (%)	16 (53.33)	17 (56.67)	33 (55.00)	0.795
	Female (%)	14 (46.67)	13 (43.33)	27 (45.00)	
Age (years)	Mean (SD)	8.62 (2.67)	8.81 (3.53)	8.71 (3.11)	0.820
	Median (min, max)	7.99 (5.08, 14.19)	8.21 (5.03, 17.40)	8.10 (5.03, 17.40)	

**Young Children cohort**		**n = 30**	**n = 30**	**n = 60**	
Gender	Male (%)	16 (53.33)	12 (40.00)	28 (46.67)	0.301
	Female (%)	14 (46.67)	18 (60.00)	32 (53.33)	
Age (years)	Mean (SD)	4.70 (0.22)	4.69 (0.19)	4.70 (0.20)	0.869
	Median (min, max)	4.74 (4.16, 4.99)	4.74 (4.27, 4.99)	4.74 (4.16, 4.99)	

### 3.2 Safety follow-up

No serious adverse event was reported during the 42 days after vaccination. Eight adverse events were reported during the follow-up period: 3 in BI group and 5 in PI group, including fever, injury, vomiting, allergic reaction and common cold, all unrelated to vaccine administration (Table 2).

**Table 2:** Adverse events in study participants after vaccination with Shanchol™ oral cholera vaccine.

After 1st dose	Adults (n = 120)		5 to <18 years (n = 60)		4 to <5 years (n = 60)	
	BI Group (n = 61)	PI Group (n = 59)	BI Group (n = 30)	PI Group (n = 30)	BI Group (n = 30)	PI Group (n = 30)
Fever	1	0	0	0	0	1
Cut injury	0	0	1	1	0	0
Vomiting	0	1	0	0	0	0
Allergic Reaction	0	0	0	0	0	0
Common cold	0	0	0	0	1	0

After 2nd dose	Adults(n = 116)		5 to <18 years (n = 60)		4 to <5 years (n = 57)	

BI Group (n = 59)	PI Group (n = 57)	BI Group (n = 30)	PI Group (n = 30)	BI Group (n = 29)	PI Group (n = 28)

Fever	0	0	0	0	0	1
Cut injury	0	0	0	0	0	0
Vomiting	0	0	0	0	0	0
Allergic Reaction	0	0	0	1	0	0
Common cold	0	0	0	0	0	0

**Total No. of adverse events**	1	1	1	2	1	2

### 3.3 Vibriocidal antibody response

**Fig. 2:**
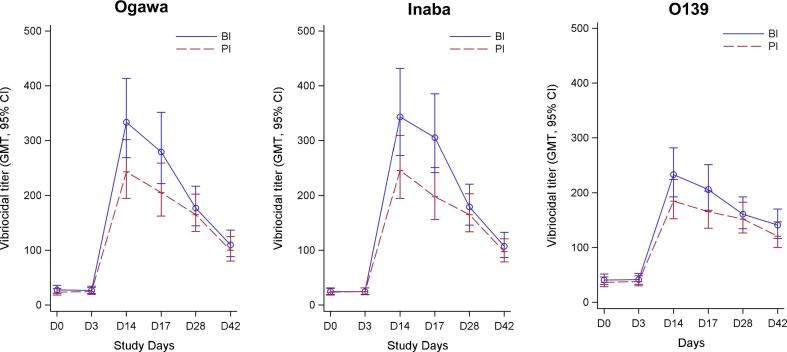
Plasma vibriocidal antibody responses in boosted (BI) and primary (PI) immunized group against different serotypes in all ages (A: O1 Inaba; B: O1 Ogawa; C: O139). The straight line () indicates the boosted (BI) immunized group and dotted line () indicates primary immunized group (PI) at different days before (D0) and after (D3, D14, D17, D28, D42) vaccination. The points indicate geometric mean titre (GMT) of vibriocidal antibody response and the standard error of mean.

The baseline vibriocidal geometric mean titres (GMTs) in the BI group (Fig. 2, Table 3) among adults, older children, young children were 57.57, 17.82 and 10.24 against Ogawa and 50.00, 16.25 and 9.09 against Inaba, respectively. We observed similar baseline vibriocidal titers in BI and PI group. There was a significant increase of vibriocidal antibody responses post first vaccine dose (day 14) in all age groups for Ogawa, Inaba and O139 in both BI and PI groups based on 95% CI (Table 3). Post second vaccine dose (day 28), GMT did not increase significantly compared to day 14 though significantly higher compared to day 0 in all age groups.

**Table 3:** Vibriocidal antibody responses by age groups after vaccination with Shanchol™ oral cholera vaccine.

	Baseline		Day 14 (14 days post first vaccine dose)		Day 28 (14 days post second vaccine dose)	
	Boosted Group (n = 59)	Primary Immunized Group (n = 57)	Boosted Group (n = 59)	Primary Immunized Group (n = 57)	Boosted Group (n = 57)	Primary Immunized Group (n = 56)
Adults	**GMT**^a^**(95% CI)**	**GMT**^a^**(95% CI)**	**GMT**^a^**(95% CI)**	**GMT**^a^**(95% CI)**	**GMT**^a^**(95% CI)**	**GMT**^a^**(95% CI)**
O1 Inaba	50.00 (34.43, 72.62)	40.98 (28.04, 59.91)	298.22 (227.26, 391.34)	273.21 (207.22, 360.22)	194.37 (150.65, 250.77)	199.93 (154.61, 258.53)
O1 Ogawa	57.57 (39.08, 84.82)	40.49 (27.30, 60.06)	268.30 (209.13, 344.20)	230.44 (178.85, 296.91)	174.22 (134.84, 225.09)	160.00 (123.56, 207.19)
O139	52.41 (36.97, 74.29)	56.23 (39.42, 80.19)	154.46 (116.85, 204.17)	176.35 (132.77, 234.24)	133.32 (100.76, 176.41)	164.01 (123.64, 217.55)

Older children	**(n = 30)**	**(n = 30)**	**(n = 30)**	**(n = 30)**	**(n = 30)**	**(n = 28)**
O1 Inaba	16.25 (9.58, 27.56)	17.82 (10.50, 30.23)	422.24 (261.50, 681.79)	393.97 (243.99, 636.13)	183.79 (123.95, 272.53)	204.94 (136.31, 308.12)
O1 Ogawa	17.82 (10.41, 30.49)	18.66 (10.90, 31.93)	473.95 (292.57, 767.78)	412.60 (254.70, 668.39)	179.59 (115.49, 279.29)	195.04 (123.49, 308.05)
O139	30.31 (19.09, 48.13)	40.00 (25.19, 63.51)	272.21 (185.21, 400.08)	201.59 (137.16, 296.28)	171.48 (123.76, 237.62)	160.00 (114.15, 224.26)

Young children	**(n = 29)**	**(n = 29)**	**(n = 29)**	**(n = 28)**	**(n = 28)**	**(n = 28)**
O1 Inaba	9.09 (5.88, 14.04)	10.49 (6.79, 16.20)	369.34 (215.10, 634.21)	118.88 (68.57, 206.09)	148.55 (87.98, 250.81)	90.54 (53.63, 152.87)
O1 Ogawa	10.24 (6.72, 15.62)	9.76 (6.40, 14.89)	360.62 (221.05, 588.31)	152.27 (92.53, 250.57)	181.08 (113.51, 288.88)	148.55 (93.12, 236.98)
O139	33.84 (21.30, 53.76)	13.97 (8.80, 22.20)	457.99 (328.10, 639.30)	185.62 (132.19, 260.64)	220.74 (158.66, 307.12)	124.91 (89.78, 173.79)

GMTs are significantly different if their respective 95% confidence intervals are non-overlapping.

a Geometric mean titres.

**Fig. 3:**
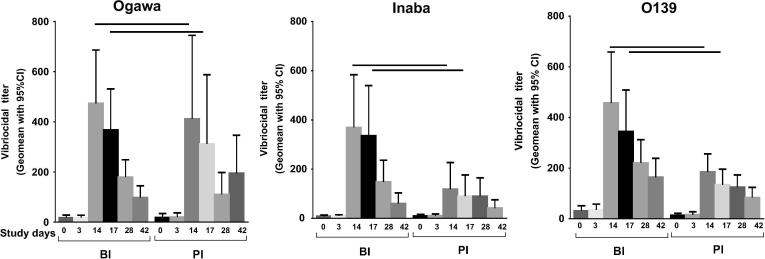
Vibriocidal plasma antibody responses. Responses were shown in children under 5 years of age who previously received a single dose of OCV 3 years earlier and revaccinated with two doses of OCV, comparatively with children who did not receive OCV earlier.

Interestingly, a significant boosting response post first dose (day 14) was seen in young children in the BI group to Inaba [GMR 95% CI: 3.36 (1.62, 6.94)], Ogawa [GMR 95% CI: 2.36 (1.17, 4.77)], and O139 [GMR 95% CI: 2.11 (1.28, 3.46)] compared to PI group (Fig. 3 and Supplementary Table 1). However, no boosting effect was seen at day 3 and day 17 for both groups. However, no boosting effect was observed post second dose (day 28).

### 3.4 LPS antigen antibody response

**Fig. 4:**
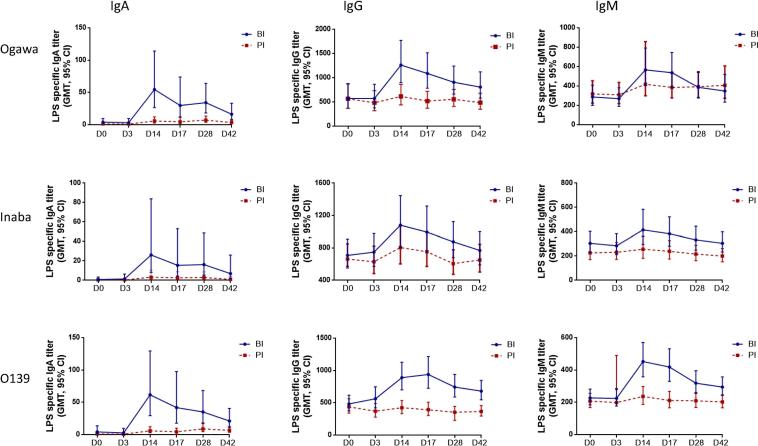
Lipopolysaccharide (LPS)-specific plasma antibody responses in young children. Immunoglobulin A (IgA), IgG and IgM antibody responses were measured against Ogawa, Inaba and O139.

IgA and IgG antibody responses against Ogawa and Inaba LPS increased significantly post first dose in all age groups for both BI and PI groups (Fig. 4, Supplementary Figs. 1 and 2). Both adults and older children developed significant IgM responses post first dose (day 14) for both BI and PI groups. However, there was no significant IgM response in young children for both BI and PI groups. In adults and older children there was no boosting effect in BI group compared to PI group at any time point. In contrast, in young children there was a significant increase of antibody responses of LPS-specific IgA (GMT: Ogawa: 54.5 vs. 5.81; O139: 62 vs. 5.8), IgG (Ogawa: 1235.7 vs. 615; O139: 914.6 vs. 423.0) and IgM (O139: 463.7 vs. 263.4) in BI group compared to PI group post first dose of vaccination (Fig. 4).

### 3.5 Memory B-cell response

We prioritized assessing LPS-specific IgG and IgA over IgM memory B-cell responses due to limited amount of PBMCs (Supplementary Fig. 3). We did not observe any significant differences in LPS-specific IgA and IgG memory B-cell responses between baseline and post vaccination at day 42 in any age group in either BI (0.02%) or PI (0.03%) groups.

### 3.6 LPS-specific antibody response in feces

**Fig. 5:**
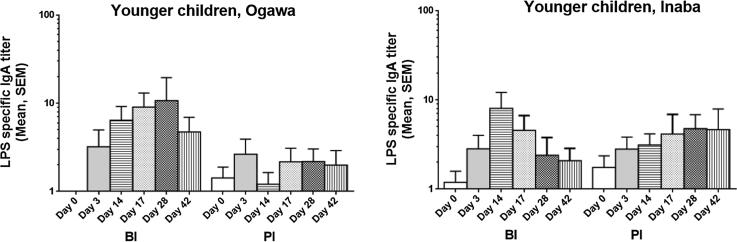
LPS-specific IgA antibody responses. Responses were observed in fecal extracts in young children against LPS Ogawa and Inaba.

Ogawa and Inaba LPS-specific IgA antibodies were assessed in fecal samples obtained from young children. In the BI group, LPS-specific Ogawa responses were significantly increased post first and second vaccine doses compared to baseline, persisting until day 42, but the increased response to Inaba for both BI and PI was not significantly different (Fig. 5). LPS-specific fecal IgA responses post first dose tended to be lower in PI group in Ogawa compared to BI group, although it was not statistically significant.

## 4 Discussion

Although children bear a heavy burden of cholera globally [23], vaccination with OCV of young children (<5 years) confers lower levels of protective efficacy and shorter duration of protection than in older children and adults [11], [24], [25], [26], [27]. However, the duration of protection after natural infection seems to be similar in children and adults suggesting that protective immunity conferred by natural infection may differ from that seen after vaccination [25]. The mechanisms behind these observations are not well understood. We have previously shown that immune responses, including memory B-cell responses, to V. cholerae LPS were associated with protection against cholera [19], [28] and that in children memory B-cell responses targeting LPS were induced and more sustained by natural infection than by vaccination [20]. Considerable effort is being directed toward developing effective mucosal vaccines appropriate delivery systems, especially those targeted to the intestine.

Our study results show comparable safety profile as observed earlier [8], [29]. We showed that immune responses measured by vibriocidal antibody assay and LPS-specific plasma antibody titers induced by OCV vaccination are stronger in participants who received a single dose boosted later by two doses compared to participants who did not receive a single dose of OCV earlier.

The baseline vibriocidal titre was significantly lower in young children compared to older children and adults. In high cholera endemic countries, higher baseline titers among adult and adolescent participants are most likely due to repeated prior exposure to *V. cholerae* [30]*.* Furthermore, the BI group had received one dose of OCV earlier, possibly boosted by repeated exposure. In young children, the lower baseline vibriocidal titers suggest that they may be less exposed to *V. cholerae* infection. In addition, young children who had received an earlier single dose of OCV (BI group) did not show a B cell memory response to LPS. In an earlier study, we have been able to show poor memory B cell responses to the LPS antigen but strong responses to cholera toxin B subunit antigen when the OCV (Dukoral) was administered [17]. However good responses is seen in cholera patients to both LPS and the toxoid antigens [16]. Thus, it is understandable that young children given the OCV, Shanchol, do not mount memory B cell responses to LPS in this study too.

There was a significant increase of vibriocidal antibody responses after the intake of a OCV dose in adults, older children and young children in both BI and PI group while post second dose of vaccine, there was a significant reduction of antibody response compared to the first immunization (day 14 vs. days 28 and 42) in all age cohorts. This may be due to the reason that antigens in Shanchol™ induced higher serum vibriocidal responses after the first dose of vaccine, reaching a maximum level that could not be boosted further by a second dose administered after a short interval [5]. Another study conducted in an urban slum of Kolkata [4] showed similar results and this was explained by the reasoning that immune response in the gut that had been stimulated by the first dose may neutralize the effects of the second dose of the oral vaccine, therefore resulting in decreased vibriocidal titers, corroborating similar findings in other studies [8], [31], [32] as well. The first booster dose of the vaccine may have elicited memory immune responses resulting in a brisk rise in vibriocidal titers with no further rises after the second dose. Therefore, a second dose of vaccine 14 days later does not increase the immunogenicity to OCV in any age group based on our earlier published data [8], [9]. But this result is different from the earlier studies using the OCV, Dukoral, which contains recombinant B subunit of cholera toxin [33] where serum vibriocidal titers increased further post second dose [34], [35]. As we have already stated, this may be due to the antigenic composition of the vaccine with higher LPS content in Shanchol™ compared to Dukoral studied earlier [4].

The boosting effect seen to the OCV in this study in children primed with a single dose earlier may not be applicable to non-endemic countries with immunologically cholera naïve people in humanitarian crisis or natural disasters where cholera outbreaks can occur suddenly.

Our data suggest that young children in endemic countries who are vaccinated with a single dose of OCV, three years earlier (BI group) showed a boosting responses to all three LPS antigens (Ogawa, Inaba and O139) after intake of a dose of vaccine in comparison to the children who received the vaccine series for the first time (PI group). As the baseline vibriocidal antibodies and memory response to *V. cholerae* are lower among the young children in both groups, the boosting effect in the BI group that led to higher vibriocidal titres is due to the earlier single dose priming. Thus, lower antigen exposure to *V. cholerae* in the PI group resulted in relatively lower immune responses than in the BI group. Thus even a single dose given over 3 years ago can act as a priming dose to further booster effect later. However, the level of protection conferred by such late boosts as well as the optimal doses needed for primary and the booster dose needs to be studied in an effectiveness trial in a cholera endemic area.

We also observed strong LPS-specific antibodies post first dose as reported earlier [20]. In addition, we observed that all 3 isotypes of antibodies (IgA, IgG and IgM) were significantly upregulated in the systemic circulation after intake of OCV three years later in comparison to the primary immunization group. However, the memory B cell (MBC) responses to LPS antigens 28 days post first vaccination was relatively poor. Earlier studies showed a similar phenomenon to OCV [20]. However, for the first time we have carried out this study to evaluate the post boost MBC response in comparison to primary immunization. However, memory response to other cholera antigens (OSP, TcpA, sialidase etc) as well as at different time intervals between priming and boosting may help to better understand B-cell memory responses in different age groups in OCV recipients.

We analyzed fecal IgA antibody responses in the study participants. The analysis shows that fecal extracts of older children and adults contained low levels of total IgA (<10 ug/mL) and so could not be further analyzed for vaccine-specific IgA responses. In contrast, half of the samples from young children fulfilled the inclusion criteria and could be analyzed for LPS specific antibodies. Low levels of total IgA in feces has also been observed in other studies recently [36]. We only observed higher responses in BI group to Ogawa LPS in the young children but not to the Inaba LPS. The antibody level to Inaba LPS in the PI group was high and not different from that seen in the BI group. It may also be mentioned that *V. cholerae* O1 Inaba was in circulation in the community at that time (unpublished data) and possibly led to higher exposure and mucosal immune responses to Inaba LPS.

Vibriocidal antibodies are believed to act as a surrogate marker and is considered as an indirect correlate of protection and used commonly to assess vaccine-induced immunity to OCVs [37]. The assays however does not provide information on vaccine efficacy following a boosting vs. primary vaccination. Similarly, the immune response measured 14 days after the second OCV dose does not effectively measure the magnitude of the immune response [17]. This study was conducted in an area where *V. cholerae* exposure is frequent. Our immunogenicity findings may not be comparable with the immune responses in non-endemic cholera-prone areas or non-endemic populations in humanitarian crisis. However, in a primed population, significantly elevated levels of vibriocidal and anti-LPS specific antibodies both in the systemic as well as in mucosal compartments were seen in young children receiving the booster dose. The results of this study also suggest that young children less than 5 years of age, need to be given booster OCV doses after an interval of around 3 years, while for older children and adults a longer interval may be sufficient similar to that observed earlier [9]. Our earlier data has shown that in an endemic setting [11] a single primary dose of OCV was not protective in young children. Based on this evidence, two doses need to be given among the young children in the primary series for protection against cholera. However, further studies are needed to determine the number of primary or booster doses as well as the appropriate interval for boosting immune responses to obtain protection in young children. Efficacy studies need to be complemented with these immunogenicity studies in an endemic population.

## Declaration of Competing Interest

The authors declare that they have no known competing financial interests or personal relationships that could have appeared to influence the work reported in this paper.

## Appendix A Supplementary material

Supplementary Figure 1 Lipopolysaccharide-specific plasma antibody responses in adults.

Supplementary Figure 2 Lipopolysaccharide-specific plasma antibody responses in older children.

Supplementary Figure 3 Memory B-cell responses by age group.
