# Supplementary figures and images for: Augmented immune responses to a booster dose of oral cholera vaccine in Bangladeshi children less than 5 years of age: Revaccination after an interval of over three years of primary vaccination with a single dose of vaccine
Source: Vaccine. 2020 Feb 11;38(7):1753–61. doi: 10.1016/j.vaccine.2019.12.034 (PMC7014297; doi:10.1016/j.vaccine.2019.12.034)

## Slide 1
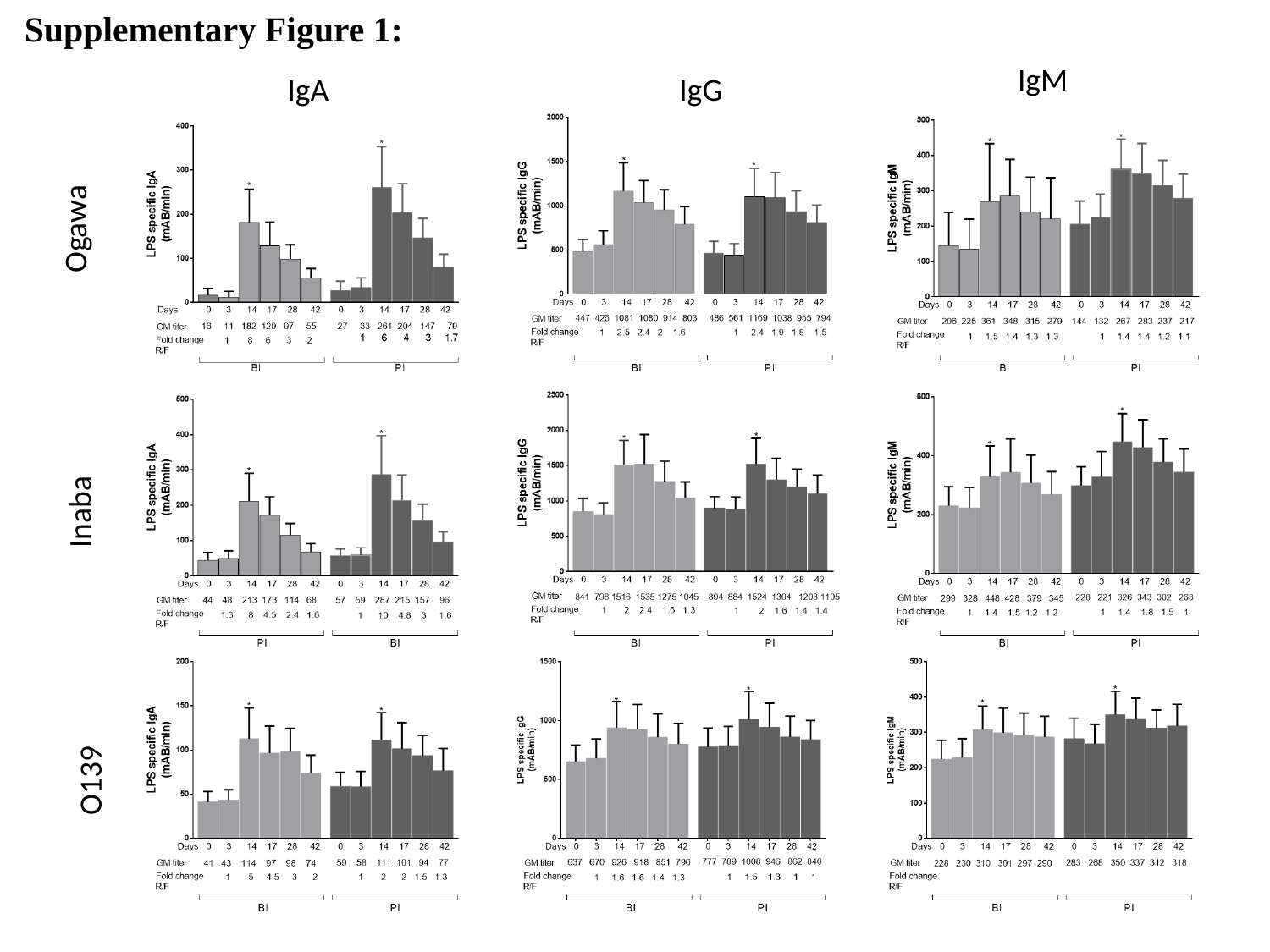

Supplementary Figure 1:
IgM
IgA
IgG
Ogawa
Inaba
O139

Supplement: Supplementary Figure 1 — Lipopolysaccharide-specific plasma antibody responses in adults. [file mmc1.pptx]

## Slide 1
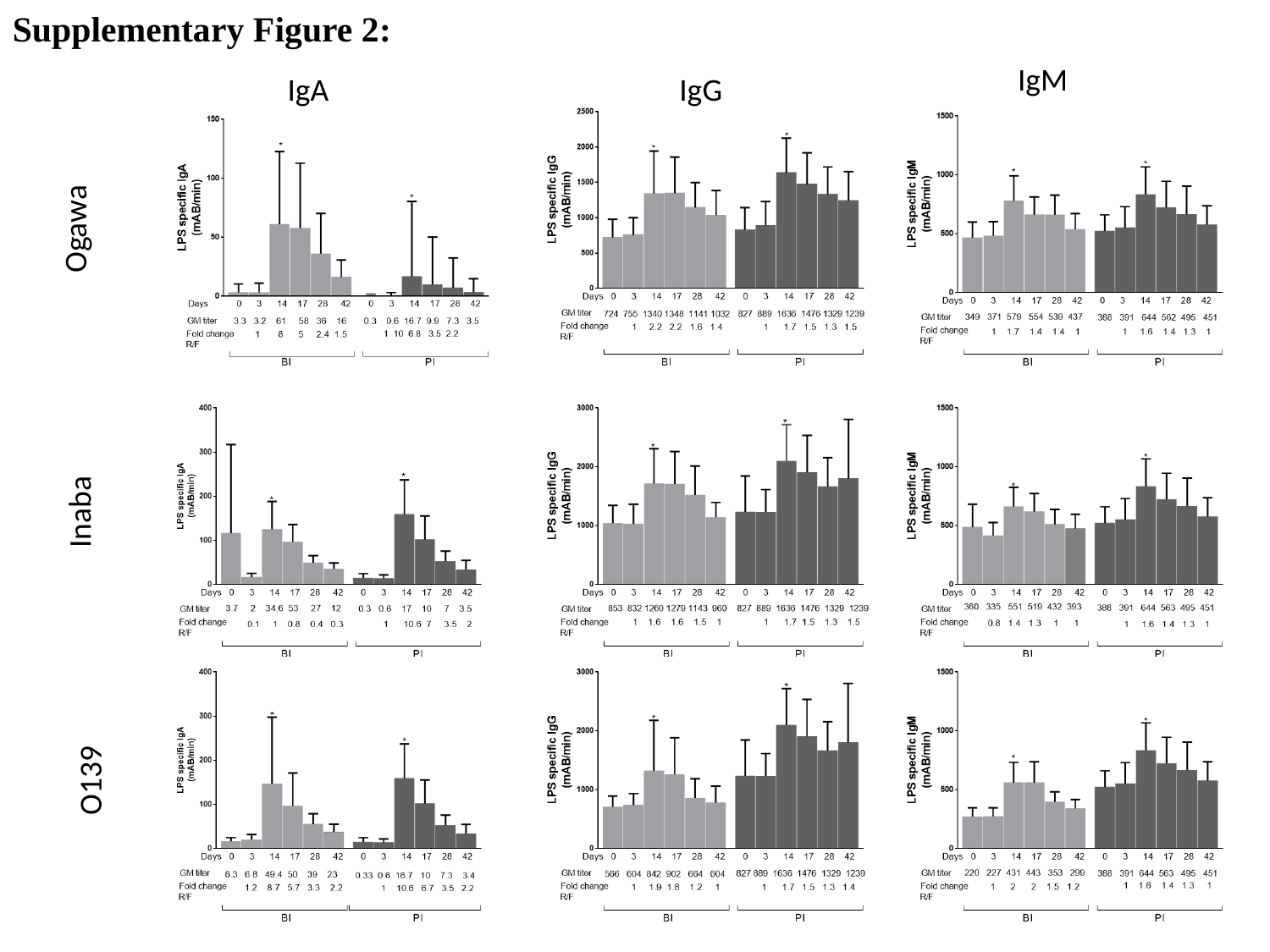

Supplementary Figure 2:
IgM
IgA
IgG
Ogawa
Inaba
O139

Supplement: Supplementary Figure 2 — Lipopolysaccharide-specific plasma antibody responses in older children. [file mmc2.pptx]

## Slide 1
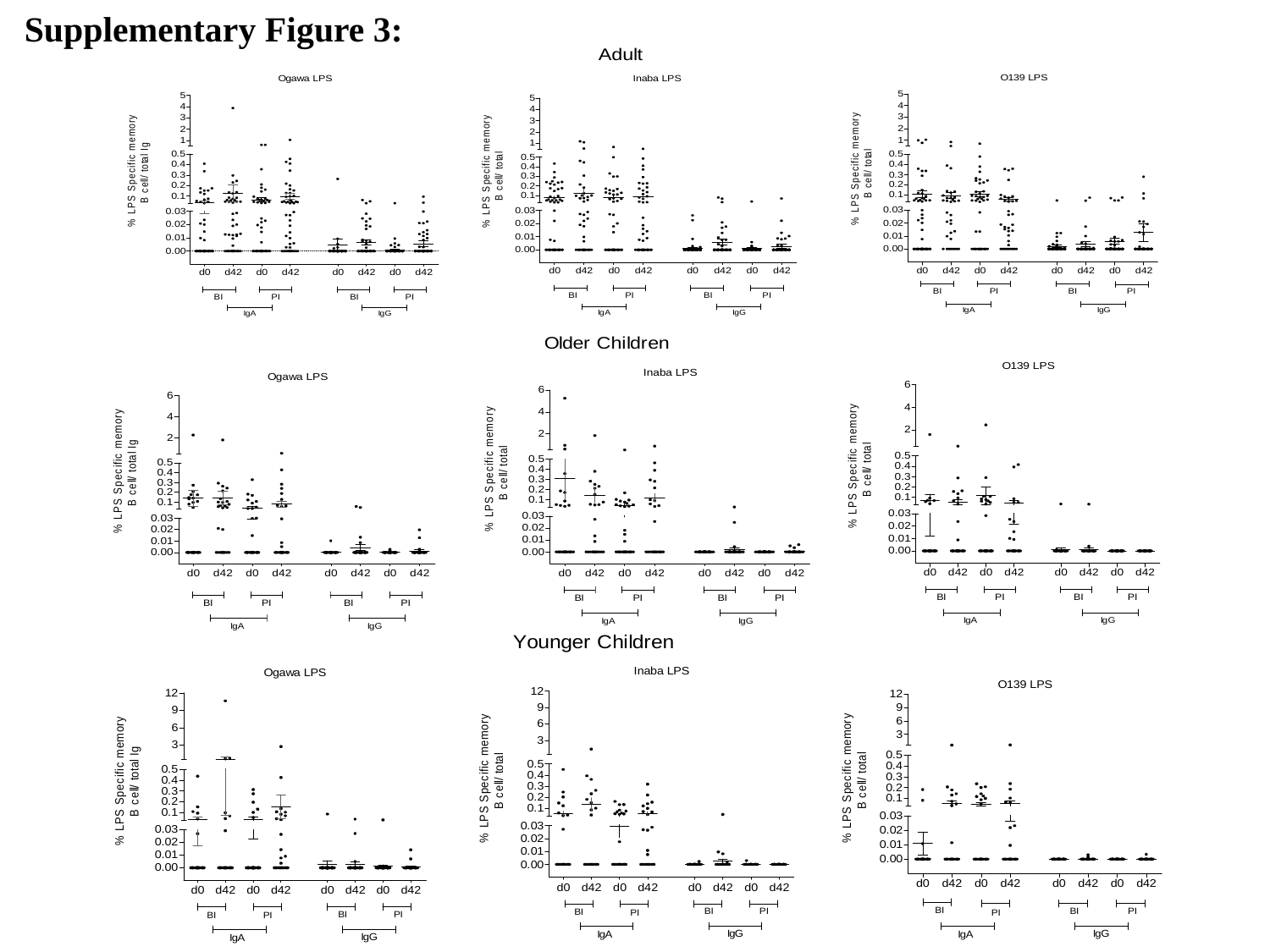

Supplementary Figure 3:

Supplement: Supplementary Figure 3 — Memory B-cell responses by age group. [file mmc3.pptx]
