# Supplementary material for: Augmented immune responses to a booster dose of oral cholera vaccine in Bangladeshi children less than 5 years of age: Revaccination after an interval of over three years of primary vaccination with a single dose of vaccine
Source: Vaccine. 2020 Feb 11;38(7):1753–61. doi: 10.1016/j.vaccine.2019.12.034 (PMC7014297; doi:10.1016/j.vaccine.2019.12.034)
Supplement: Supplementary Table 1 [file mmc4.docx]

**Supplementary Table 1: Vibriocidal antibody responses in young children**

| **Younger children** | **Day 14**  **(14 days post first vaccine dose)** | **Day 28**  **(14 days post second vaccine dose)** |
| --- | --- | --- |
|  | **GMR^¶^ (95% CI)** | |
| **O1 Inaba** | 3.36 (1.62, 6.94) | 1.78 (0.90, 3.53) |
| **O1 Ogawa** | 2.36 (1.17, 4.77) | 1.22 (0.64, 2.32) |
| **O139** | 2.11 (1.28, 3.46) | 1.30 (0.84, 2.01) |

¶ Adjusted for baseline Geometric mean titers and age strata in all ages model. Geometric mean ratio (GMR) of GMTs of Boosted group to Primary immunized group
